# Management of Congenital Morgagni Hernia With Gastric Outlet Obstruction Causing Perforated Esophagus in a Young Adult

**DOI:** 10.1016/j.atssr.2025.06.028

**Published:** 2025-07-25

**Authors:** Nicole Lin, Merit Gorgy, Kenji Okumura, Falyn Katzman, Matthew McGuirk, Clara Angeles, Tracey Weigel

**Affiliations:** 1 Department of Surgery, Westchester Medical Center, New York Medical College, Valhalla, New York; 2 Department of Surgery, University of Wisconsin School of Medicine and Public Health, Madison, Wisconsin

## Abstract

This report presents a rare case of esophageal perforation in a patient with a large, incarcerated Morgagni hernia. At presentation, the Morgagni hernia contained peritoneal contents, including the stomach and duodenum. Because of distal obstruction resulting in persistent emesis, the patient presented in a septic state in the setting of esophageal perforation. We present a hospital course where the esophageal perforation was repaired on an emergency basis through a left thoracotomy, followed by a delayed robotic-assisted diaphragmatic repair using mesh.

This case report discusses a rare and challenging occurrence of concurrent Boerhaave syndrome in the setting of a large, incarcerated congenital Morgagni hernia. Both conditions are rare and morbid in thoracic surgery, and their simultaneous presentation requires complex clinical judgment and surgical management.^1^, ^2^, ^3^ Patients presenting with sepsis have a high risk of morbidity and mortality. We detail a single hospital course where the esophageal perforation was repaired on an emergency basis, followed by a delayed diaphragmatic repair with mesh by using a minimally invasive technique. This case provides multiple distinctive learning points, thus making it particularly worthy of discussion. This patient has authorized release of this case for publication.

A 35-year-old man with a past medical history of asthma, hypertension, type 2 diabetes, and obesity (body mass index, 38 kg/m^2^) presented with nausea, vomiting, and abdominal and chest pain to an outside facility approximately 1.5 hours from our quarternary center. At the time of arrival to our emergency department, he had experienced symptoms for approximately 72 hours. He was normotensive but pale, dyspneic, tachycardic, and diaphoretic. Initial laboratory results were remarkable for a leukocyte count of 30 × 10^9^/L. A chest roentgenogram showed a bilateral pleural effusion with right lower lobe opacity and chest wall emphysema. Computed tomography of the thorax showed esophageal perforation with extraluminal contrast material in the distal mediastinum with pneumomediastinum and bilateral pleural effusions. In addition, there was a large right Morgagni-type hernia containing distal stomach, duodenum, and colon (Figure 1). The patient received a diagnosis of perforated esophagus secondary to emesis from a gastric outlet obstruction caused by a congenital Morgagni hernia.

**Figure 1:**
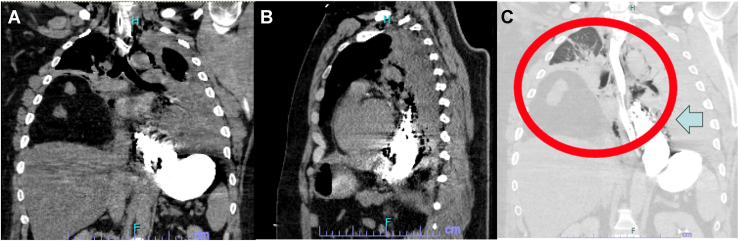
(A, B) Computed tomography showing an esophageal leak and (C) a Morgagni diaphragmatic hernia filling the right hemithorax. The circle and arrow show extravasation of oral contrast material in the distal esophagus and pneumomediastinum.

The patient was taken to the operating room on an emergency basis. After timeout, flexible esophagoscopy was performed. There was an obvious large perforation at approximately 32 to 38 cm from the incisors. The esophagoscope was pulled back, and a nasogastric tube and postpyloric feeding tube were inserted. Bronchoscopy was performed, with copious amounts of clot extracted. Next, a bronchial blocker was positioned for isolation of the right lung. The patient was positioned in a right lateral decubitus position, with his arms in the praying position with beanbag mattress support. We incised along the top of the seventh rib by using a Tuffier retractor to spread the muscles. On entering the chest, we found copious amounts of bilious, bloody fluid. There was a black hernia sac overlying the esophagus. We were able to isolate the esophagus below the aortopulmonary window and placed Penrose drains proximal and distal to the defect. This maneuver helped elevate the esophagus, with an obvious 3-inch vertical perforation noted in the left lateral wall of the esophagus (Figure 2). We peeled back some of the necrotic hernia sac to expose the defect. We extended the myotomy of the muscle to delineate the defect. The mucosa was then closed in an interrupted fashion using 4-0 polyglactin 910 suture (Vicryl, Ethicon), and the esophageal muscle layer was closed using 4-0 silk suture. A drain was placed posterior to the esophagus. The epicardial fat pad was fashioned into a flap to cover the esophageal repair—tacked posteriorly to the pleura, inferiorly to the edge of the crus, and anteriorly to the hernia sac. The chest was thoroughly irrigated with multiple liters of saline solution. Esophagoscopy was performed again, with no evidence of a leak. At this time, given his critical condition, we brought the patient to the intensive care unit for resuscitation.

**Figure 2:**
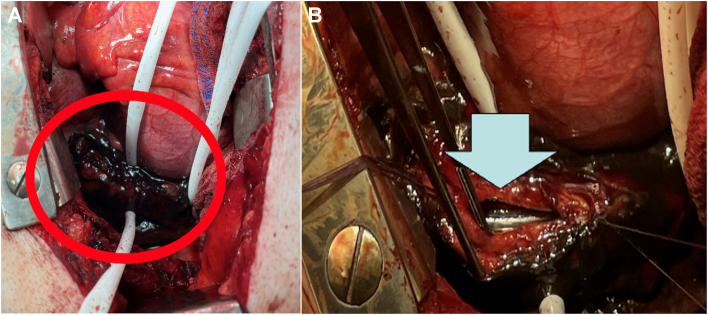
(A) Perforated esophagus (circle) where (B) the nasogastric tube (arrow) can be seen in the distal esophagus.

On postoperative day 4, the patient underwent computed tomography of his chest and abdomen that confirmed an intact repair. On postoperative day 6, an esophagram further confirmed a durable repair. Enteral feeding was initiated. On postoperative day 10, he was taken back to the operating room for reduction and repair of the diaphragmatic hernia. We reduced the abdominal contents from the Morgagni hernia without injury to nearby structures (Figures 3A to 3C). After reduction, the hernia sac was incised along the edge of the diaphragm defect. We avoided entering the right and left pleural space in this operation, given the contamination. Expanded polytetrafluoroethylene mesh (Gore-Tex Dualmesh, W. L. Gore & Associates) was placed along the inferior and lateral edge, sewn with 0 polyester (Ethibond, Ethicon) running and interrupted sutures to buttress the repair. The anterior edge was sewn by making approximately 5 small incisions in the skin of the upper abdomen and using a fascial closure device to place 0 Ethibond along the anterior edge of the defect (Figures 3D to 3F). The patient tolerated the procedure well and was discharged home 5 days later. One year later, the patient is doing well and has returned to his employment.

**Figure 3:**
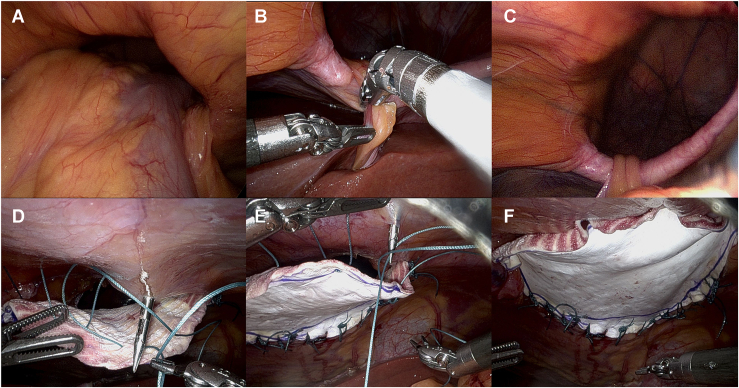
(A-C) Reduction of Morgagni hernia with robotic-assisted thoracic surgery. (D-F) Patch repair of large Morgagni hernia.

## Comment

This case report presents a rare presentation and subsequent management of this disease. Esophageal perforation in the setting of a congenital Morgagni hernia is largely unreported.^4^ Morgagni hernia represents 2% to 5% of all congenital diaphragmatic hernias and is usually asymptomatic and found incidentally,^5^ It is 1 of 4 types of diaphragmatic hernias and is in the anterior, retrosternal location of the chest. Repair is usually recommended, especially in symptomatic patients, because there is a 10% risk of bowel obstruction, strangulation, and volvulus.^6^ Both abdominal and chest approaches are accepted, with mesh recommended for hernias larger than 20 cm.^7^ In our patient, the diaphragmatic defect allowed peritoneal contents to herniate into the chest, with resulting partial gastric outlet obstruction leading to persistent emesis and esophageal rupture.

Early intervention is key to prevent morbidity and mortality in Boerhaave syndrome, which has a mortality rate of 60% with intervention and higher than 90% without intervention.^8^ Esophageal perforation in the setting of a diaphragmatic defect represents a complex case because the patient is critically ill, with decreased respiratory and physiologic capacity for a prolonged operation. Cases often involve large incisions, sometimes requiring both thoracotomy and laparotomy, which can be morbid and painful for the patient. In addition, a contaminated surgical field would prohibit repair or likely require the use of a less durable biologic mesh repair of a large defect. In our case, we were able to reduce and repair the diaphragmatic hernia in a less contaminated field by approaching from the abdomen, thereby avoiding pleural violation, along with providing an opportunity for a durable repair using expanded polytetrafluoroethylene. Delayed repair of the diaphragmatic hernia allowed use of a robotic-assisted minimally invasive technique in a more controlled setting. Overall, early intervention of the esophageal perforation followed by strategic delayed repair of the diaphragmatic hernia from an abdominal approach allowed for an optimal outcome in this highly lethal disease.
